# Systematic Literature Review: Professional Situation of Gifted Adults

**DOI:** 10.3389/fpsyg.2022.736487

**Published:** 2022-05-18

**Authors:** Maren Schlegler

**Affiliations:** Faculty of Economics and Business, Business Education, Goethe-University Frankfurt, Frankfurt, Germany

**Keywords:** giftedness, gifted adults, literature review, professional situation, stereotypes

## Abstract

A person's intelligence level positively influences his or her professional success. Gifted and highly intelligent individuals should therefore be successful in their careers. However, previous findings on the occupational situation of gifted adults are mainly known from popular scientific sources in the fields of coaching and self-help groups and confirm prevailing stereotypes that gifted people have difficulties at work. Reliable studies are scarce. This systematic literature review examines 40 studies with a total of 22 job-related variables. Results are shown in general for (a) the employment situation and more specific for the occupational aspects (b) career, (c) personality and behavior, (d) satisfaction, (e) organization, and (f) influence of giftedness on the profession. Moreover, possible differences between female and male gifted individuals and gifted and non-gifted individuals are analyzed. Based on these findings, implications for practice as well as further research are discussed.

## Introduction

In general, there is a (positive) relationship between intelligence and various aspects of the occupation, in particular occupational choice, occupational status, occupational performance, and occupational satisfaction. Individuals with a higher IQ choose more challenging and socially recognized occupations (Gottfredson, 2003; Schmidt and Hunter, 2004; Herrnstein and Murray, 2010) and intelligence correlates most strongly positively with investigative as well as weakly positively with realistic and weakly negatively with social occupational interest (Sparfeldt, 2006; Pässler et al., 2015). Furthermore, positive correlations of intelligence with occupational status, income, and occupational success are shown (Gottfredson, 2003). Individuals with higher intelligence are more likely to advance professionally and earn higher incomes (Schmidt and Hunter, 2004; Schmidt, 2009). Numerous meta-analyses show a positive relationship between intelligence and job performance (Hunter, 1986; Schmidt and Hunter, 1998; Gottfredson, 2003; Kuncel et al., 2004; Schmidt, 2009; Wai, 2014; Schmidt et al., 2016; Murtza et al., 2020), where the strength of the relationship may be overestimated due to the methods used (Richardson and Norgate, 2015). Meta-analyses show a higher correlation during training (*r* = 0.63) than after training (*r* = 0.55), but intelligence remains the best predictor of job performance even with increasing work experience (Schmidt and Hunter, 2004). A higher expression of general intelligence appears to lead to higher performance in all occupations. Thus, general intelligence predicts occupational performance better than special talents as well as non-cognitive factors such as occupational interest and other personality traits (Hunter, 1986; Ree et al., 1994; Schmidt and Hunter, 1998, 2004; Gottfredson, 2003; Schmidt et al., 2016). Additionally, intelligence correlates positively with job satisfaction in most studies (Wulff et al., 2009; Thompson and Lane, 2014; Murtza et al., 2020).

Highly intelligent or gifted individuals should therefore have a high level of professional success. This is often doubted, especially in the advice literature. There is talk of difficult career biographies (Schwiebert, 2015), problems with colleagues (Schwertfeger, 2013), emotional hypersensitivity (Lovecky, 1986), and general job dissatisfaction (Plucker and Levy, 2001). If the negative assumptions about the occupational situation of gifted employees were true, this would be problematic from a resource-oriented point of view, because the potential of the employees underlying the giftedness would not be sufficiently utilized in the occupation (Kinzelmann, 2013). This would be extremely unsatisfactory for both the organizations and the gifted individuals themselves. However, in both earlier and current studies and publications of giftedness research, the focus is predominantly on children and adolescents and their support at school (Urban, 2001; Meier et al., 2014; Jost, 2020a,b). Scientific evidence on gifted people in early adulthood is available in vocational training and studies (Stamm, 2004, 2005, 2006; Stamm and Niederhauser, 2008; Badel, 2014). Accordingly, gifted adolescents are more interested in intellectual-research occupations and less interested in social occupations (Sparfeldt, 2006, 2009). However, a few studies show that these interests later manifest themselves in career choices and that gender differences exist concerning employment situations (Schlegler et al., 2018). On the other hand, there are numerous popular scientific publications and guidebooks on gifted adults and especially on the professional situation (vom Scheidt, 2006; Dietrich, 2007; Brackmann, 2010, 2015; Hussla, 2010; Groll, 2011; Trappe, 2011; Lackner, 2012; Heintze, 2013; Kinzelmann, 2013; Reiblein, 2013; Schwertfeger, 2013; Schwiebert, 2015). But empirical studies are rare (Fabio and Buzzai, 2020). To the author's knowledge, only one comprehensive review exists on gifted adults (Rinn and Bishop, 2015). That review examines all aspects of adult life, including the personality of gifted individuals, development after school depending on the school support measures experienced, and family life. Statements on the occupational situation are only made for the occupational success and occupational satisfaction variables; other variables are not analyzed (Rinn and Bishop, 2015).

One reason for this lacuna could be that no standard definition of giftedness exists (Gagné, 1993; Sparfeldt, 2006; Rost, 2010; Fabio and Buzzai, 2020). First, a distinction is made between the different polarities of giftedness. Static concepts assume that giftedness exists from birth to the end of life (giftedness as an unchangeable characteristic). An adult with excellent performance would thus already have the corresponding predisposition in childhood in the sense of a high intelligence quotient (IQ), which does not change over time (Ziegler, 2007). Dynamic concepts, on the other hand, postulate the idea that giftedness changes with age (Baudson, 2016) and depends significantly on the support and the associated learning processes of the individual (Ziegler, 2007). Complementing this categorization, giftedness definitions can be divided into four classes: Competency definitions, performance or *post-hoc* definitions, unidimensional definitions, and multidimensional definitions (Preckel et al., 2012). The most significant distinction seems to be between giftedness as competence or performance. Competence understands giftedness as a disposition or as a latent capacity. Someone is gifted if he or she has an extremely high development potential, which, however, does not have to express itself in behavior. A person can achieve top performance but does not have to do so (Holling and Kanning, 1999; Freeman, 2001; Mönks and Katzko, 2005; Preckel et al., 2012). Most definitions are based on the factor analytical model of intelligence, in which a distinction is made between a general factor (g) and several special factors (s) of intelligence (Spearman, 1904). g is understood as the ability to acquire declarative and procedural knowledge quickly and effectively, to apply it adequately in varying situations, to learn from the experience gained, and to recognize to which other situations the knowledge gained can be transferred and to which it cannot (Rost, 2009). Based on this, giftedness is often defined quantitatively: A person is gifted if his or her IQ is at least two standard deviations above the mean (i.e., IQ 130 or percentile rank 98). Assuming a normal distribution of intelligence, it follows that 2% of the population is gifted (Rost and Sparfeldt, 2017).

Performance, on the other hand, assumes that giftedness is observable. Only someone is gifted who performs or has already performed recognizably and is far above average (Holling and Kanning, 1999; Mönks and Katzko, 2005; Preckel et al., 2012). The understanding of giftedness as performance has, e.g., been presented in the Munich Model of Giftedness. It understands giftedness as a multidimensional construct of giftedness factors (predictors), moderating non-cognitive personality traits and environmental characteristics, and performance domains in which high performance can occur. Accordingly, giftedness develops through the interaction of factors internal and external to the person. High performance can only be achieved if both favorable giftedness factors and non-cognitive personality traits, as well as a nurturing environment, are present in sufficient expression (Heller and Perleth, 2007). Other models that understand giftedness as performance include the Talent Development Mega-Model (Subotnik et al., 2011), and the Talent-Development-Achievement-Domains (TAD) Framework (Preckel et al., 2020). In the Talent Development Mega-Model, giftedness is understood as a dynamic concept, with giftedness in adults determined by performance and in children and adolescents determined by potential (Subotnik et al., 2011). Based on this understanding, gifted children and adolescents (defined by competency definition) would not necessarily grow up to be gifted adults unless they translate their ability into peak performance (Preckel and and Vock, 2021). This leads to the existence of underachievers, who have a high potential for exceptional performance but do not translate this into high performance. In this case, the observed performance (e.g., school performance or professional performance) deviates significantly from the performance expected based on the giftedness negatively (Scarr, 1994; Sparfeldt, 2006; Gagné, 2012; Sparfeldt et al., 2014).

Analogous to the Marburg Giftedness Project (Rost, 2009), this review considers only academic and intellectual giftedness. This kind of intelligence can be measured by intelligence tests (Preckel, 2010), school achievement tests such as the Scholastic Assessment Test (which has a high correlation with general intelligence; Frey, 2019), or by academic and occupational performance (Sparfeldt, 2006; Rost, 2013). Other types of giftedness, such as athletic and musical talent as described in, e.g., the Munich Model of giftedness, were excluded. In the German-speaking context, gifted individuals refer to the definition of potential and high achievers refer to the definition of performance (Wirthwein, 2010). In the Anglo-American context, on the other hand, gifted people are defined based on outstanding performance (according to the performance definition), and gifted people designated in the German context (according to the potential definition) are referred to as highly intelligent people (Subotnik et al., 2011). To present a picture as broad as possible of the professional situation of gifted individuals, such differentiation is deliberately omitted here.

This paper aims to provide a systematic literature review of the occupational situation of gifted adults. The results on the employment situation, job satisfaction, and differences due to gender or giftedness, which have mostly been available in popular science, shall be replaced by scientifically based findings. Besides, this review is also intended to present the subjective experiences of a group and to counter the stereotypes of gifted people that are prevalent in the majority of society—the tendency of gifted individuals to have emotional and social problems (Baudson, 2016). Finally, this review will identify existing research gaps and develop ideas for future research approaches.

## Article Types

The present work is a systematic literature review (Daigneault et al., 2014; Booth et al., 2016) and was conducted based on the PRISMA reporting standards (Moher et al., 2009). The search was conducted in German and English on May 16, 2019, in the EBSCOhost (Business Source Premier, EconLit with Full Text, PsycInfo, and PsycArticles), Web of Science, WISO, and ProQuest databases. No restrictions were set about publication dates. According to PICOS, the search terms included population (gifted adults), outcome (occupation), and study designs (all types of methods). The following search terms (German and English) were used: hochbegab^*^ AND (beruf^*^ OR arbeit^*^ OR karriere^*^ OR job^*^ OR beschäftig^*^), and gifted^*^ AND (job^*^ OR vocation^*^ OR occupation^*^ OR work^*^ OR profession^*^ OR career^*^). Search terms such as talent, intelligence, men and women, and the use of Google Scholar were deliberately omitted because an initial overview search returned a very large number of articles (stopping after 35,000 hits) as well as results on millennials, artificial intelligence, talent management, and gender equity in the workplace. After discussing the search terms with other researchers and analyzing articles in giftedness research, the search terms were reduced to those listed above. By not selecting the population (adults), articles that also examine the career choice interests of gifted adolescents are expected. However, these can be excluded from further analysis. The search of the databases was supplemented by a snowball search. This involved evaluating the bibliographies of all included articles and searching for additional hits.

**Figure 1:**
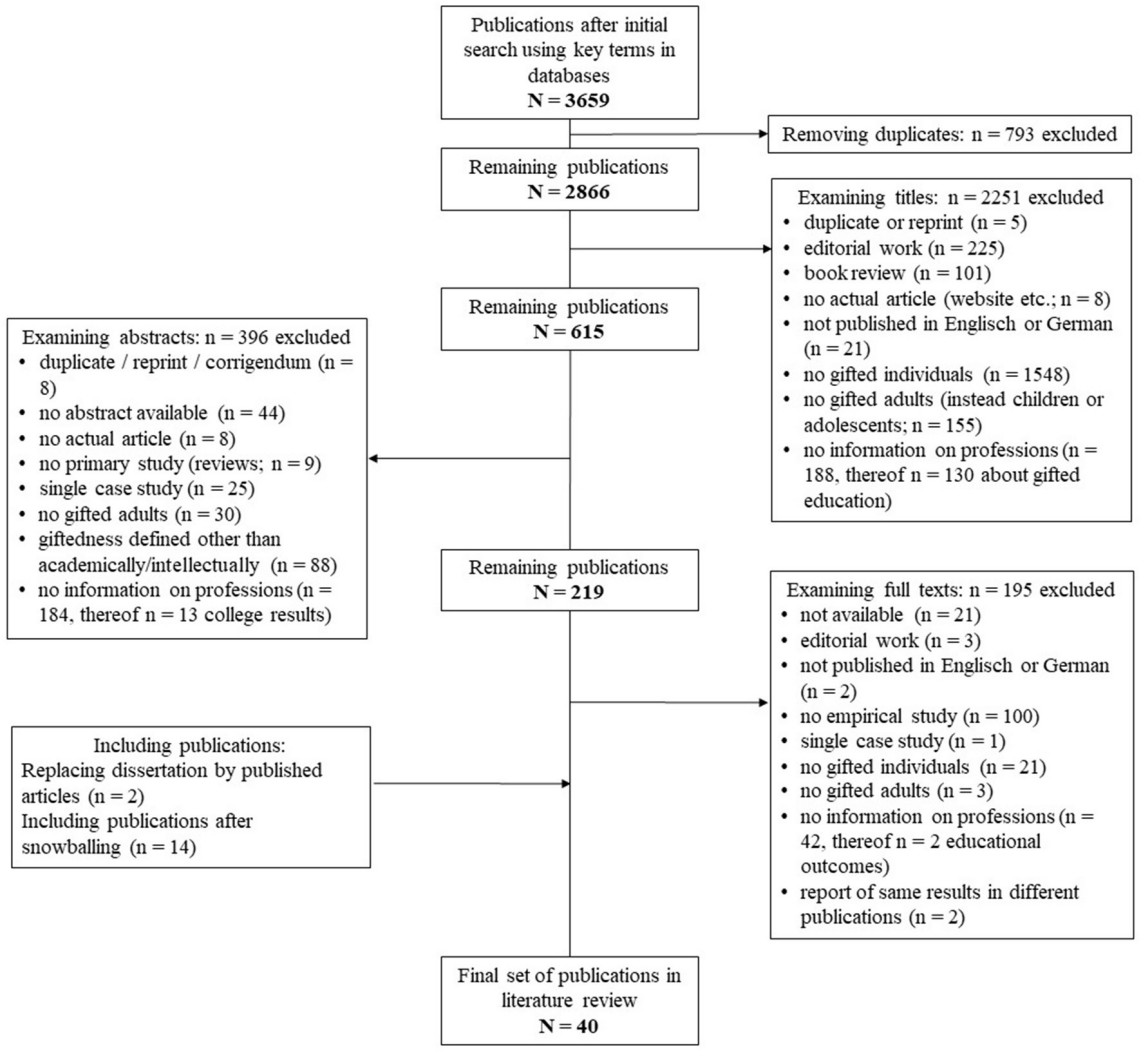
PRISMA flow diagram of the publication selection process.

Figure 1 illustrates the selection process. The database search yielded 3,658 hits. These were matched systematically and in different steps with the following inclusion criteria:

1. it is an empirical primary study with a sample *N* > 1;
2. the population under consideration is explicitly referred to by the authors as gifted and giftedness refers to intelligence or academic achievement (other fields such as sports, music, and creativity were excluded);
3. the professional situation after the studies or training is analyzed; and
4. the publication is written in English or German.

Initially, 793 duplicates were removed (see Figure 1). The remaining 2,865 publications were analyzed by title; 2,251 articles were excluded. Of the remaining 614 results, abstracts were analyzed and another 396 articles were excluded. Finally, 218 full texts were examined in the last step. One dissertation was replaced by three individual partial publications from the dissertation due to lack of retrievability (Schuster, 1986). Twenty-eight articles met all criteria and are included in the review. These were supplemented with 14 articles from the snowball search. Two publications reported the same results as other included publications and are therefore not included (Miller and Kastberg, 1995; Webb et al., 2002). Thus, 40 publications are included in the review.

The 40 studies included are 26 journal articles (peer-reviewed), nine contributions to collective works, three monographs, one journal, and one dissertation. The majority of publications (*n* = 27) originated from larger projects (see also Table 1). Twenty-eight studies were quantitative surveys, six were qualitative studies, and six used a mixed-methods design. Twenty-one studies used the performance definition, while 19 studies used the potential definition of giftedness. The distribution based on the definition types is balanced. Table 2 provides a more detailed overview of the characteristics of each study, such as the location and timing of data collection, the sample, and the definitional category used (competence or performance) to identify the gifted.

**Table 1:** Description of included studies.

**Characteristic**	**Specification**
Region	*n* = 34 (85%) United States, *n* = 6 (15%) Europe [thereof: *n* = 2 (5%) Germany, *n* = 1 (2.5%) Austria/Germany, *n* = 1 (2.5%) Poland, *n* = 1 (2.5%) Sweden, *n* = 1 (2.5%) Finland]
Time frame of data collection	*n* = 29 (72.5%) longitudinal studies, *n* = 11 (27.5%) cross-sectional studies
Time of data collection	*n* = 6 (15%) before 1990, *n* = 4 (10%) 1990–1999, *n* = 5 (12.5%) 2000–2009, *n* = 3 (7.5%) after 2010, missing data *n* = 12 (30%)
Projects	*n* = 13 (32.5%) Terman study, *n* = 7 (17.5%) Study of mathematically precocious youths (SMPY), *n* = 2 (5%) UCLA Giftedness program, *n* = 1 (2.5%) Presidential scholars, *n* = 1 (2.5%) CHOICE, *n* = 1 (2.5%) Illinois valedictorian project, *n* = 1 (2.5%) Marburg giftedness project, *n* = 1 (2.5%) Westinghouse science talent search, *n* = 13 (32.5%) single studies
Sample size	6–3,398 Gifted individuals [<100 *n* = 9 (22.5%), 101–999 *n* = 22 (55%), >1,000 *n* = 9 (22.5%)]
Gender	54.79% male and 45.21% female (no data on other genders), missing data *n* = 1 (2.5%); in *n* = 1 sample 100% male, in *n* = 11 samples 100% female
Age	Report of average values *n* = 22 (55%; 24–85 years), report of spans *n* = 9 (22.5%; 18–90 years), report of median *n* = 1 (2.5%; 49 years), approximate data *n* = 2 (5%; 32–33 years), missing data *n* = 10 (25%)
Definition of giftedness	Giftedness as performance *n* = 21 [52.5%, thereof^a^: *n* = 4 (10%) SAT-M, *n* = 5 (12.5%) SAT-M or SAT-V, *n* = 4 (10%) Year's best school/university, *n* = 4 (10%) academic competition, *n* = 2 (5%) matriculation top university, *n* = 3 (7.5%) national educational test, *n* = 2 (5%) professional success (publications/awards)]; Giftedness as potential *n* = 19 [47.5%, thereof: *n* = 18 (45%) criterion IQ minimum score 130–140 points or percentile rank 98, *n* = 1 (2.5%) other giftedness indicators]

**Table 2:** Included studies with information on the region, data collection, sample, and definition of giftedness used.

**References**	**Sample**	**Data collection**	**Project**	**Region**	**Definition of giftedness**	
					**Potential**	**Performance**
Arnold, 1993	*n* = 82	1981–1991	Illinois Valedictorian Project	United States		Year's best school
Benbow et al., 2000	*n* = 1975	1999	SMPY	United States		SAT-M >390 points (at age 13)
Feist, 2006	*n* = 161	Missing data	Single study	United States		Academic competition
Ferriman et al., 2009	*n* = 879	1992, 2003/2004	SMPY	United States		Matriculation from a top university; SAT-M ≥ 700 points or SAT-V ≥ 630 points (at age 13)
Holahan, 1981	*n* = 352	1977	Terman study	United States	IQ ≥ 135	
Holahan, 1985	*n* = 102	1940, 1972	Terman study	United States	IQ ≥ 135	
Holahan, 1994	*n* = 414	1972–1977	Terman study	United States	IQ ≥ 135	
Holahan, 2003	*n* = 185	1940, 1950, 1972, 1996	Terman study	United States	IQ ≥ 135	
Holahan et al., 1999	*n* = 383	1960, 1972, 1992	Terman study	United States	IQ ≥ 135	
Holahan and Sears, 1995	*n* = 1,063	1972, 1977, 1982, 1986	Terman study	United States	IQ ≥ 135	
Hollinger and Fleming, 1992	*n* = 126	1984	CHOICE	United States	Other giftedness indicators	
Hossiep et al., 2012	*n* = 496 (and n = 2,654 non-gifted)	2012	Single study	Germany	IQ ≥ 130	
Kastberg and Miller, 1996	*n* = 6	Missing data	Single study	United States	Year's best school	
Kaufmann et al., 1986	*n* = 139	Missing data	Presidential scholars	United States	National educational test	
Kell et al., 2013	*n* = 320	2003/2004, 2008/2009	SMPY	United States	SAT-M ≥ 700 points and/or SAT-V ≥ 630 points (at age 13)	
Lubinski et al., 2014	*n* = 1,650	2012/2013	SMPY	United States	SAT-M	
Lubinski et al., 2006	*n* = 966	2003/2004	SMPY	United States	SAT-M ≥ 700 points and/or SAT-V ≥ 630 points (at age 13); matriculation from a top university	
Makel et al., 2016	*n* = 259	2012–2014	Single study	United States	SAT-M ≥ 700 points and/or SAT-V ≥ 630 points (at age 13)	
Oden, 1968	*n* = 1,188	1960	Terman study	United States	IQ > 140	
Park et al., 2008	*n* = 1,586	Missing data	SMPY	United States	SAT-M	
Perrone et al., 2004	*n* = 113	2001/2002	Single study	United States	Year's best school, national educational test	
Persson, 2009	*n* = 287	2007	Single study	Sweden	IQ > 130	
Pollet and Schnell, 2017	*n* = 339 (and *n* = 136 non-gifted)	Missing data	Single study	Austria, Germany	IQ ≥ 98th percentile	Year's best university
Reis, 1996	*n* = 12	Missing data	Single study	United States		Professional success
Schuster, 1990	*n* = 35	1984/1985	UCLA Giftedness program	United States		National educational test
Schuster, 1993	*n* = 28	1990	UCLA Giftedness program	United States		National educational test
Sears and Barbee, 1977	*n* = 430	1972	Terman study	United States	IQ ≥ 135	
Sears, 1977	*n* = 486	1972	Terman study	United States	IQ ≥ 135	
(Shareef, 2015)	*n* = 322	Missing data	Single study	United States	Identified as gifted in school	
Siekańska and Sekowski, 2006	*n* = 90 (and *n* = 90 non-gifted)	Missing data	Single study	Poland		Academic competition
Subotnik et al., 1989	*n* = 156	Missing data	Single study	United States	IQ ≥ 140	
Subotnik et al., 1999	*n* = 19	Missing data	Westinghouse Science Talent Search	United States		Academic competition
Terman and Oden, 1959	*n* = 1,528	1940, 1945	Terman study	United States	IQ ≥ 135	
Terman and Oden, 1967	*n* = 1,528	1950–1955	Terman study	United States	IQ ≥ 135	
Tirri and Koro-Ljungberg, 2002	*n* = 11	Missing data	Single study	Finland		Professional success; academic competition
Tomlinson-Keasey, 1990	*n* = 40	1936, 1945, 1951, 1972, 1977	Terman study	United States	IQ ≥ 135	
Tomlinson-Keasey and Keasey, 1993	*n* = 657	1920–1999	Terman study	United States	IQ ≥ 135	
Wai et al., 2005	*n* = 3,398	1992–2003	SMPY	United States		SAT-M ≥ 390 points or SAT-V ≥ 370 points; SAT-M ≥ 500 points or SAT-V ≥ 430 points; SAT-M ≥ 700 points or SAT-V ≥ 630 points
Wai et al., 2010	*n* = 1,467	Missing data	SMPY	United States		SAT-M ≥ 500 points; SAT-M ≥ 700 points
Wirthwein and Rost, 2011	*n* = 101 (and n = 91 non-gifted)	2006/2007	Marburg giftedness project	Germany	IQ ≥ 130 (3rd grade) and IQ ≥ 125 (9th grade)	

In this systematic review of the literature, we categorized the analyzed variables into the following thematic areas: (a) employment situation, (b) career, (c) personality and behavior, (d) satisfaction, € organization, (f) influence of giftedness on the profession, and (g) miscellaneous.

In analyzing the included studies, the author first worked out which job-related variables were examined in each case. Variables that are not related to the occupational situation (e.g., satisfaction with family life or marriage) are not considered because they do not correspond to the focus of the review. When studies examine the same or similar variables, these results are combined. The variables may have been collected by different instruments. For example, occupational status is examined in nine studies using a variety of instruments: Hollingheads Level 1–7 (Hollinger and Fleming, 1992), the adapted version of the Minnesota Occupational Scale (Terman and Oden, 1959, 1967), and self-developed instruments (Tomlinson-Keasey and Keasey, 1993). To make the results comparable, the mean values given were rescaled. The new mean value can take on values from 0 to 1. The higher the value, the greater the expression of the characteristic. Where status groups (occupational status) are indicated, 1 denotes the highest status group. Hence 2, etc., then denote the lower status groups in descending order. To make the results on the occupational fields comparable, categories were formed and the individual occupations or occupational fields mentioned in the respective study were classified there (an adapted version of Bundesagentur für Arbeit, 2011). The respective categories for the studies are only reported if they are also mentioned in the study. However, it is then unclear whether there are no mentions for the category or whether it falls under the frequently used category “other”, which is not reported here due to its low informative value. For the data on women, only women who are in paid employment are considered. If housewife was specified as an occupation in the study, the data were corrected accordingly by this factor. For income, only results where group comparisons by gender and presence of giftedness were included in the analyses are evaluated. Other results are not reported, as these would be distorted by the different survey dates, because income levels have risen sharply in absolute terms over the last century (OECD, 2020). In longitudinal studies, the individual publications are considered individually, because even within longitudinal overall studies, the samples differ between the individual publications in terms of size and composition.

In each section, results for each of the variables examined are first described. Then, where reported, any differences between gifted females and gifted males and gifted and non-gifted individuals are described.

## Results

### Employment Situation

The employment situation contains information about the forms of employment, occupational status, income, working hours and work preferences.

In general, the employment situation is often described by the categories of employed and non-employed and, in the case of women, often into part-time and full-time employment. Accordingly 83–85% of the gifted individuals are employed (Pollet and Schnell, 2017), while for the women, 83% are employed more than half the usual weekly hours, 8.5% are not employed at all (Schuster, 1990), and 75% are employed full time (Schuster, 1993). In the study by Shareef (2015) nearly half (43.6%) of the gifted sample hold positions that involve managing personnel. In the study by Pollet and Schnell (2017), high achievers are more likely to hold a management position than gifted (highly intelligent) individuals (74 vs. 35%). This could already be due to the way the sample was obtained because the high achievers were selected based on their present academic performance, that is, they already translate their intelligence into high academic or professional performance. The gifted individuals, on the other hand, are members of Mensa, an association of highly intelligent gifted individuals (IQ ≥ 130). They have demonstrated their potential, but do not necessarily have to deliver an actual performance. As expected, the work experience (in years) of gifted individuals varies and depends on age (Shareef, 2015).

Table 3 shows the occupational fields in which gifted men and women work. The proportions in the occupational fields differ greatly among the individual studies. The ranges are small if the occupational fields have a low share in all studies (religion, security, agriculture, forestry, and horticulture) or are only mentioned in one study (transportation and logistics). Larger ranges show up in the science, technology, engineering, and mathematics (STEM) and research and development occupational fields. One reason for this seems to be the selection and promotion of the respective sample. Feist (2006) sample (88% in research and development), for example, are finalists in a national science competition, thus showing a very high affinity as well as performance in this field already at school age. For women in STEM careers, the data differ greatly between the Study of Mathematically Precocious Youths (SMPY) studies and the Terman studies, but within the longitudinal studies, they are similar. This may presumably be due to the timing of data collection (much later for SMPY and thus a different role model for women) and the selection and strong promotion of SMPY participants. In the social professions, the range is significantly wider for women than for men. The proportion of men in social professions is consistently low, while for women it also depends on the sample selection and the time of data collection. Occupational fields with a low proportion of women are transport and logistics and security occupations, which are traditionally considered male-dominated occupations (OECD, 2007; Bundesministerium für Arbeit und Soziales, 2017; Bundesamt für Güterverkehr, 2019; Ng and Acker, 2020). Overall, the proportion of gifted workers appears to be highest in management, organization and commerce, STEM, healthcare, and research and development.

**Table 3:** Occupational fields in which gifted men and women work.

**Occupational field (examples occupations)**	**Sample**	**Percentage**	**References**
**Agriculture, forestry, and horticulture (farmers, foresters, gardeners)**			
	Men	1.2–1.6%	Terman and Oden, 1959**, Terman and Oden, 1967**; Oden, 1968**
	Women	0.4–1.7%	Holahan, 1994**; Holahan and Sears, 1995**
**Arts (artists, authors, journalists, directors, producers, photographers, graphic designers)**			
	Men and women	2.6–6.1%	Wai et al., 2005*; Persson, 2009; Kell et al., 2013*; Makel et al., 2016
	Men	2.3–12.2%	Terman and Oden, 1959**, Terman and Oden, 1967**; Oden, 1968**; Subotnik et al., 1989; Schuster, 1990***; Benbow et al., 2000*; Lubinski et al., 2014*
	Women	2.6–13.4%	Terman and Oden, 1959**, Terman and Oden, 1967**; Oden, 1968**; Subotnik et al., 1989; Schuster, 1990***; Holahan, 1994**; Holahan and Sears, 1995**; Benbow et al., 2000*; Lubinski et al., 2014*
**Craft (mason, carpenter, joiner)**			
	Men and women	0.4–14.1%	Wai et al., 2005*; Persson, 2009; Kell et al., 2013*
	Men	2.7–5.0%	Terman and Oden, 1959**, Terman and Oden, 1967**; Oden, 1968**; Subotnik et al., 1989
	Women	1.2–3.0%	Subotnik et al., 1989; Schuster, 1990***
**Healthcare (physicians, nurses, pharmacists, veterinarians)**			
	Men and women	3.9–36.8%	Kaufmann et al., 1986; Arnold, 1993; Subotnik et al., 1999; Wai et al., 2005*; Persson, 2009; Kell et al., 2013*; Makel et al., 2016
	Men	5.0–25.7%	Oden, 1968**; Subotnik et al., 1989; Benbow et al., 2000*; Lubinski et al., 2006*; Lubinski et al., 2014*; Terman and Oden, 1959**, Terman and Oden, 1967**
	Women	0.8–15.9%	Terman and Oden, 1959**, Terman and Oden, 1967**; Oden, 1968**; Subotnik et al., 1989; Schuster, 1990***, 1993***; Holahan, 1994**; Holahan and Sears, 1995**; Benbow et al., 2000*; Tirri and Koro-Ljungberg, 2002; Lubinski et al., 2006*; Lubinski et al., 2014*
**Law and economics (judges, lawyers, prosecutors, economists)**			
	Men and women	0.4–13.0%	Kaufmann et al., 1986; Arnold, 1993; Wai et al., 2005*; Siekańska and Sekowski, 2006; Kell et al., 2013*; Makel et al., 2016;
	Men	3.4–20.3%	Terman and Oden, 1959**, Terman and Oden, 1967**; Oden, 1968**; Subotnik et al., 1989; Benbow et al., 2000*; Lubinski et al., 2006*; Lubinski et al., 2014*
	Women	0.8–9.1%	Terman and Oden, 1959**, Terman and Oden, 1967**; Oden, 1968**; Subotnik et al., 1989; Schuster, 1990***, 1993***; Holahan, 1994**; Holahan and Sears, 1995**; Benbow et al., 2000*; Lubinski et al., 2006*; Lubinski et al., 2014*
**Management, organization, and commerce (businessmen, entrepreneurs, office activities)**			
	Men and women	29.2–34.6%	Arnold, 1993; Wai et al., 2005*; Persson, 2009; Makel et al., 2016
	Men	17.5–38.4%	Terman and Oden, 1959**, Terman and Oden, 1967**; Oden, 1968**; Subotnik et al., 1989; Benbow et al., 2000*; Lubinski et al., 2006*; Lubinski et al., 2014*
	Women	15.1–43.8%	Terman and Oden, 1959**, Terman and Oden, 1967**; Oden, 1968**; Subotnik et al., 1989; Schuster, 1990***, 1993***; Holahan, 1994*; Holahan and Sears, 1995*; Kastberg and Miller, 1996; Benbow et al., 2000*; Lubinski et al., 2006*; Lubinski et al., 2014*
**Religion (clergy, employees in religious communities)**			
	Men and women	0.6–0.8%	Wai et al., 2005*; Makel et al., 2016
	Men	0.5–1.2%	Terman and Oden, 1959**, Terman and Oden, 1967**; Oden, 1968**
	Women	0.5–1.2%	Terman and Oden, 1959**; Schuster, 1990***, 1993***
**Research and development (researchers, university teachers, professors)**			
	Men and women	5.1–88.3%	Kaufmann et al., 1986; Subotnik et al., 1999; Wai et al., 2005*; Feist, 2006; Siekańska and Sekowski, 2006; Persson, 2009; Kell et al., 2013*; Makel et al., 2016
	Men	5.8–41.1%	Terman and Oden, 1959**, Terman and Oden, 1967**; Oden, 1968**; Subotnik et al., 1989; Benbow et al., 2000*; Lubinski et al., 2006*; Lubinski et al., 2014*
	Women	3.8–72.3%	Terman and Oden, 1959**, Terman and Oden, 1967**; Oden, 1968**; Subotnik et al., 1989; Schuster, 1990***, 1993***; Holahan, 1994**; Holahan and Sears, 1995**; Kastberg and Miller, 1996; Benbow et al., 2000*; Tirri and Koro-Ljungberg, 2002; Lubinski et al., 2006*; Lubinski et al., 2014*
**Security professions (firefighters, police, military)**			
	Men	0.6–1.4%	Wai et al., 2005*; Makel et al., 2016
	Women	2.6–3.3%	Terman and Oden, 1959**, Terman and Oden, 1967**; Oden, 1968**
**Social services (teachers, educators, social workers, librarians)**			
	Men and women	1.3–5.2%	Arnold, 1993; Wai et al., 2005*; Persson, 2009; Kell et al., 2013; Makel et al., 2016
	Men	0.7–5.5%	Terman and Oden, 1959**, Terman and Oden, 1967**; Oden, 1968**; Subotnik et al., 1989; Benbow et al., 2000*; Lubinski et al., 2006*; Lubinski et al., 2014*
	Women	1.9–37.2%	Terman and Oden, 1959**, Terman and Oden, 1967**; Oden, 1968**; Subotnik et al., 1989; Schuster, 1990***, 1993***; Holahan, 1994**; Holahan and Sears, 1995**; Kastberg and Miller, 1996; Benbow et al., 2000*; Tirri and Koro-Ljungberg, 2002; Lubinski et al., 2006*; Lubinski et al., 2014*
**STEM (scientists, engineers, technicians, architects)**			
	Men and women	5.3–31.9%	Arnold, 1993; Subotnik et al., 1999; Wai et al., 2005*; Siekańska and Sekowski, 2006; Persson, 2009; Kell et al., 2013; Makel et al., 2016
	Men	2.7–35.2%	Terman and Oden, 1959**, Terman and Oden, 1967**; Oden, 1968**; Subotnik et al., 1989; Benbow et al., 2000*; Lubinski et al., 2006*; Lubinski et al., 2014*
	Women	0.9–22.6%	Oden, 1968**; Subotnik et al., 1989; Holahan and Sears, 1995**; Benbow et al., 2000*; Tirri and Koro-Ljungberg, 2002; Lubinski et al., 2006*; Lubinski et al., 2014*
**Transportation and logistics (truck drivers, pilots)**			
	Men	0.60%	Wai et al., 2005*
	Women	0.10%	Terman and Oden, 1959**

*SMPY Project, **Terman Project, ***UCLA Giftedness Program.

The SMPY project considers differences between groups identified as gifted as children and adolescents (through the SAT) or as adults (through graduate study). To better distinguish the groups, they are further referred to as talent-search participants and graduate students, analogous to the original study. The graduate students are significantly more likely to be university lecturers, engineers, or scientists than the other group. The difference decreases by more than half when professions in medicine and law are included (Lubinski et al., 2006).

Gifted individuals are more likely to be self-employed than high achievers (Pollet and Schnell, 2017). This may indicate that gifted individuals, defined by high intelligence, are more task-oriented and, as independent workers, can shape their tasks and relationships at work.

Based on the SMPY sample, Table 4 shows how gifted individuals' work preferences change between the ages of 25 and 35 (Ferriman et al., 2009) and the gender differences in each case at age 35 (Ferriman et al., 2009) and 50 (Lubinski et al., 2014). Gifted males are significantly more likely than females to value career success, knowing that their achievements have an impact, making a lot of money (all small effects), and having a full-time career (medium effect). Gifted women are significantly more likely to value a temporary part-time career (medium effect; Benbow et al., 2000). Consistently, gifted men more strongly prefer career-related aspects (full-time career, having a lot of money, being successful at work and in their industry), while women more strongly prefer family-related aspects (part-time career, being there for family and friends, living close to family, spending time with children every day, having strong friendships; Ferriman et al., 2009; Lubinski et al., 2014).

**Table 4:** Work preferences by gender.

**Item**	**Change in preference between 25 and 35 years** ^**a**^		**Gender differences at age 35^b^**	**Gender differences at age 50^c^**
	**Men**	**Women**		
**Payment**				
Performance-based salary system	↑	↑	n.s.^d^	M > W^2^
Higher-than-average salary	n.s.	n.s.	n.s.	M > W^2^
Reasonable benefit package	n.s.	n.s.	M > W	-^e^
Good pension scheme	n.s.	n.s.	n.s.	n.s.
Health insurance benefits	-	-	-	n.s.
**Position**				
Holding an administrative position	↓	↓	n.s.	n.s.
Taking over leadership	↑	↑	n.s.	n.s.
Promotion possibility	n.s.	n.s.	n.s.	M > W^2^
Contributing to decisions	n.s.	n.s.	n.s.	M > W^1^
Work results significantly influence others	n.s.	n.s.	n.s.	W > M^1^
**Fame**				
Prestige/reputation of the organization	n.s.	n.s.	n.s.	n.s.
Prestige of the job	n.s.	n.s.	n.s.	M > W^1^
Knowing how well I work in my projects	n.s.	n.s.	n.s.	-
**Tasks**				
Varied tasks	↓	↓	n.s.	n.s.
Doing similar tasks every day	n.s.	n.s.	n.s.	-
**Skills**				
Using many complex skills	n.s.	n.s.	n.s.	-
Having the ability to do my job well	↓	↓	n.s.	W > M^1^
Possibility to learn new things	n.s.	n.s.	n.s.	n.s.
Use of skills at a high level	-	-	-	M > W^1^
**Satisfaction**				
Enjoying the work	↓	↓	W > M	n.s.
Satisfaction with the work	↓	↓	n.s.	W > M^1^
**Relationships**				
Developing friendships with people	↓	↓	n.s.	n.s.
Respecting colleagues	n.s.	n.s.	W > M	W > M^2^
Mentoring young colleagues	-	-	-	M > W^1^
Working with people	-	-	-	W > M^1^
Exchanging ideas informally with colleagues	-	-	-	n.s.
Friendly colleagues	-	-	-	n.s.

*^a^For all effects, |d| <0.22, p < 0.01*.

*^b^For all effects, |d| < 0.22, p < 0.01*.

*^c^The study was conducted with two cohorts*.

*^d^The difference was not significant*.

*^e^The item is not reported*.

*The numerical superscript indicates whether the result applies to both cohorts (2) or only one cohort (1)*.

*F, female; M, male; n.s., not significant*.

Gender differences in agreement with various work and personality-related statements have also been examined. Table 5 shows that gifted men and women rate themselves differently. If one tries to categorize these statements based on the life goals in the Aspirations Index (personal growth, relationships, society, health, wealth, fame and attractiveness; Klusmann et al., 2005), gifted men show higher agreement with the life goal fame and lower agreement with the life goal relationships than gifted women. No differences are shown in the life goals society and personal growth. No items can be assigned to the life goals health, wealth, and attractiveness.

**Table 5:** Statements on work and personality by gender (Ferriman et al., 2009).

**Variable^**c**^**	**Agreement**	
	**Talent-search participants^**a**^**	**Graduate students^**b**^**
**Fame**		
I want to be recognized as the best in my field.	M > W	M > W
**Relationships**		
I tend to put myself and my own needs before others and their needs.	M > W	M > W
It is important to me that no one goes without or gets left behind.	W >M	W >M
I am a team player.	n.s.	n.s.
**Community**		
I want to improve the human condition.	n.s.	n.s.
Society has a responsibility to meet the basic need of all its members.	n.s.	n.s.
I think that people have a duty to provide for those less fortunate than themselves.	n.s.	n.s.
I believe that the most important contribution one can make to humanity involves the direct improvement of others‘ lives.	n.s.	n.s.
I make a contribution to the greater good.	n.s.	n.s.
**Personal development**		
I have the inner strength to resist popular pressure.	n.s.	n.s.
**Miscellaneous**		
I have the capacity for sustained physical activity, playing, and moving about, without tiring and having to rest	M > W	M > W
Society should invest in my ideas because they are more important than those of other people in my discipline.	M > W	M > W
The prospect of receiving criticism from others does not inhibit me from expressing my thoughts.	M > W	M > W
I am able to control my emotions when it is appropriate to do so.	M > W	M > W
I can relatively easily multitask or do multiple things at once.	W >M	W >M
I persist when others give up.	W > M	M > W
I can relatively easily shift gears among different tasks.	M > W	W > M
I am comfortable spending long intervals of time by myself.	n.s.	n.s.
I believe that the most important contribution one can make to humanity is the discovery of scientific principles.	n.s.	n.s.
The possibility of discomforting others does not deter me from stating the facts.	n.s.	n.s.
I approach individuals in higher ranked positions than my own (e.g., to ask questions or to discuss possible collaborations).	n.s.	n.s.
I enjoy being part of an organization where individuals share responsibilities	n.s.	n.s.
I tend to take charge and give directions	n.s.	n.s.

*^a^For all effects, |d| < 0.35, p < 0.01*.

*^b^For all effects, |d| < 0.22, p < 0.01*.

*^c^Classification of the items according to Klusmann et al. (2005)*.

*F, female; M, male; n.s.; not significant*.

In addition, other studies show that gifted men are more likely to be employed than gifted women (Terman and Oden, 1967) and are also (significantly) more likely to work full time (Terman and Oden, 1967; Lubinski et al., 2014). Additionally, gifted men wish to—and do—work significantly more hours per week than gifted women (on average 11 h more; Benbow et al., 2000; Lubinski et al., 2014). If only employed people are considered (no homemakers), men work on average 4–7 h more per week than women (Benbow et al., 2000). In consequence, gifted men earn (significantly) more than gifted women (Terman and Oden, 1959; Oden, 1968; Subotnik et al., 1989; Holahan and Sears, 1995; Benbow et al., 2000; Wai et al., 2005; Lubinski et al., 2014). The difference is smaller when only full-time employees are considered (Lubinski et al., 2014). However, some studies find no income differences between gifted men and women (Lubinski et al., 2006). Moreover, gifted men are significantly more likely to switch from scientific to non-scientific careers than gifted women. They are also significantly more likely than gifted women to pursue non-academic careers in industry and government (Feist, 2006). Lubinski et al. (2014) show that only in the subsample from the university context are there differences exist occupational status. Specifically, gifted men more often have a professorship at a large research university than gifted women (Benbow et al., 2000).

Compared with non-gifted individuals of the same gender, gifted men and women are more likely to be employed (Terman and Oden, 1959; Sears and Barbee, 1977). Furthermore, there is no difference in job tenure between gifted and non-gifted individuals, that is, gifted individuals do not change jobs less frequently or more frequently than non-gifted individuals (Siekańska and Sekowski, 2006). Gifted males can be found to have a higher occupational status than male college graduates in general, regardless of educational level (Terman and Oden, 1959, 1967). Here, the difference between gifted and non-gifted males with college degrees is particularly large (Terman and Oden, 1967). In the Terman sample, most men (94.7%) work in the two highest status groups (out of a total of six groups), and about half (51.1%) even work in the highest status group. Occupational status is more positively related to educational level, one's own ambition, and the financial/social situation of parents than to IQ (Holahan and Sears, 1995). Gifted women generally have higher occupational status than non-gifted women. In the study by Hollinger and Fleming (1992), 70.6% of the female participants belong to the two highest status groups (out of a total of seven groups). None of the women work in the three lowest groups. In the Terman sample of gifted women, compared with the population average, employed gifted women are more likely to work as professionals and managers. The difference between gifted and non-gifted women is greatest in the group of women without a college degree (Sears and Barbee, 1977). The occupational status of gifted women is most positively related to attitudes in early adulthood (high importance of occupational goals), educational level, and marital status (single; Holahan and Sears, 1995). Tomlinson-Keasey and Keasey (1993) on the other hand, show in their study that there is no difference in occupational status between gifted women with and without college degrees. However, gifted women with graduate study degrees (i.e., very high levels of education) have significantly higher occupational status than gifted women with and without college degrees.

Only one study examines the differences in occupational fields between gifted and non-gifted individuals (Siekańska and Sekowski, 2006). According to this study, there are only minor differences in the shares in research and development, STEM, and the arts (≤ 5% point differences, fewer gifted individuals). Larger differences are seen in the proportion of those working in law and economics per group (15% point differences, fewer gifted individuals) and humanities (27% point difference, more gifted individuals).

Gifted individuals (men and women) have higher incomes than comparison groups of non-gifted individuals (including population averages and college graduates; Terman and Oden, 1959; Schuster, 1990; Tomlinson-Keasey and Keasey, 1993). This difference could be explained by the higher level of education of gifted people because individuals with a higher level of education generally also have higher incomes (for Germany Anger and Geis, 2017; Institut für Arbeitsmarkt- und Berufsforschung, 2018).

Overall, it can be stated that gifted individuals are mostly employed and that if there are differences in the employment situation compared with non-gifted individuals, these differences are in favor of gifted individuals. Comparative values from the overall U.S. population show that 5.48% of the workforce held a management position in May 2019. Thus, gifted individuals appear to be significantly more likely to hold management positions than the overall population (U.S. Bureau of Labor Statistics, 2020). Both men and women show that gifted individuals have a higher occupational status than non-gifted individuals. This can be attributed to several factors, both in the individuals themselves (personality, attitudes, marital status for women) and in their background (educational level, socioeconomic status of parents). IQ does not seem to have a significant influence. The results also contradict (popular) scientific assumptions according to which gifted people change jobs more often and faster than non-gifted people (Gusovius, 2005; Schwiebert, 2015, p. 152). There are only minor differences in occupational fields between gifted and non-gifted individuals. Differences in the income between gifted and non-gifted individuals are again in favor of gifted people. The more frequent employment compared with non-gifted people could be due to the higher educational level of gifted individuals. In general, it is assumed that the level of education has a positive influence on employment (Institut für Arbeitsmarkt- und Berufsforschung, 2018; Bundeszentrale für politische Bildung, 2019).

### Career

The theme career includes information about career goals, career paths and patterns, critical incidents and compromises in professional life and the professional success.

The career goals most frequently cited by gifted people are career success or advancement (27%) and updating skills and evidence (14%; Perrone et al., 2004). In early adulthood, gifted men with higher occupational status are significantly more likely to cite career success as a life goal than those with lower occupational status (Holahan and Sears, 1995). Gifted individuals consider the most common barriers to achieving career goals to be a commitment to non-work roles (27%), organizational politics or interpersonal relationships at work (15%), lack of motivation or self-confidence (14%), or the labor market and economy (13%). To overcome these barriers, gifted individuals see their greatest support in the areas of family support (23%), professional support (18%) and the labor market (11%). Career hurdles and career success are directly and indirectly (via adaptability) related to each other (Perrone et al., 2004). Overcoming career hurdles and achieving career goals depends on the gifted person's ability to adapt, but not on the social support they experience. It is therefore up to the person him- or herself whether he or she achieves his or her career goal.

More than half of gifted individuals say they have lived up to their intellectual abilities in their careers. Gifted individuals with low career status are more likely than those with high career status to feel that they have not fulfilled their intellectual potential. For women, this difference is significant (Holahan, 2003).

Contrary to popular assumptions, the scientific discussion assumes that a portion of gifted people are very successful in their professions (Feist, 2006). When measuring professional success, a distinction is made between subjective (a person's self-assessment) and objective professional success; in the latter case, indicators vary widely among studies, but often academic achievements such as patents and publications are recorded. A large proportion of gifted people (70% of women and about 65% of men) describe themselves as successful in their careers (subjective professional success; Benbow et al., 2000). Objective career success among gifted men is significantly greater than among gifted women (Holahan, 1985). In the SMPY project, of the gifted participants, 39% have at least one peer-reviewed publication, 9% have at least one patent (Kell et al., 2013; Makel et al., 2016), and 3.1% have at least one patent in a Fortune 500 company (Park et al., 2008). Tirri and Koro-Ljungberg (2002) report for a female sample of (former) scientists that as many as 82% have at least one publication and 18% have at least one patent. For the SMPY sample, Wai et al. (2010) show that one-third of the participants demonstrate professional success in STEM fields (doctorate, publication, professorship, patent, or profession). However, gifted individuals also achieve success in the arts and humanities (e.g., prose, theater productions, paintings, business start-ups; Oden, 1968; Kell et al., 2013; Makel et al., 2016). This is noteworthy because the results of Kell et al. (2013) and Makel et al. (2016) come from the SPMY longitudinal section in which participants were promoted in STEM fields. Thus, the funding does not seem to necessarily lead to career success exclusively in STEM fields. Lubinski et al. (2006) again show differences between talent-search participants and graduate students (see also employment situation). Accordingly, male graduate students hold academic positions (professorships) more frequently than male talent-search participants. While there is generally no difference between the groups concerning professorships among women, female talent-search participants more frequently hold professorships at high-ranking institutions. Significantly more graduate students than talent-search participants hold a patent (Lubinski et al., 2006). Because the differences were examined only for the university career field, no conclusions can be drawn about differences beyond that between gifted individuals identified in adolescence or adulthood.

Gifted men show stable careers that are influenced mainly by external factors (e.g., the economic situation). Most changes tend to be of a smaller scale (minor change of field of activity) or happen in later adulthood (change to other fields of work). One-fifth of the gifted men would retrospectively make a different career decision and would rather choose a different professional field (Holahan and Sears, 1995). Gifted women show more complex career patterns than men due to their dominant role in childrearing, with periods of entry into employment, periods of no employment, and periods of re-entry into employment (Holahan, 1994). The complex career patterns are categorized differently in the studies. Table 6 shows the classification by Holahan and Sears (1995). Twenty-seven percent of career women are homemakers for at least 10 years. After that, most return to full-time employment and show a variety of patterns. Career women older than 35 years especially experience significant career changes (Holahan and Sears, 1995). Table 7 shows a further classification of women's career patterns according to Tomlinson-Keasey (1990). Overall, about two-thirds of women switch between the patterns in their lifetime (enablers have to switch when the partner dies; mothers have to re-evaluate what they do when the children are grown up), which is why no clear statements can be made about the proportions. It is reported that about 50% belong to the enabler pattern and about 15% to the partner pattern. Besides, depending on the time in their lives, 50% of the women belong to the independent worker pattern (Tomlinson-Keasey, 1990). The majority of gifted women (≥65%) are satisfied with their career paths (Sears and Barbee, 1977; Tomlinson-Keasey, 1990). Here, employed women show significantly higher satisfaction than housewives (79 vs. 62%). Retrospectively, more women would choose paid employment and fewer would choose to work as housewives (Sears and Barbee, 1977; Tomlinson-Keasey and Keasey, 1993; Holahan and Sears, 1995).

**Table 6:** Career patterns of women according to Holahan and Sears (1995).

**Career pattern**	**Description**
Homemaking	They are not gainfully employed for most of their lives. Gainful employment takes place before starting a family and is later replaced by volunteer work.
Career	They are usually employed full-time after starting a family and pursue a career.
Income work	They work to secure their livelihood (no career aspirations): 15% of them are constantly employed.

**Table 7:** Career patterns of women according to Tomlinson-Keasey (1990).

**Career type**	**Description**
The woman who enables	They have no career of their own but support their partner in all areas of life except their job. Their satisfaction comes from the success of their partner.
The woman as a mother	They focus on their children and often take leadership positions as a volunteer.
The woman as a partner	They are equally involved in their partner's work and career and continue it even when he or she leaves. Their satisfaction arises from the marriage.
**The woman as an independent worker**	
Further development of joint work	They independently develop joint work with their partner.
Work independently	They have to work independently due to difficulties in the partnership (e.g., divorce or death).
Singles	They have no partner and must support themselves.
Career-oriented	They pursue an individual career.

Among gifted women, length of employment and earnings do not correlate with career success (Schuster, 1990). Reis (1996) also notes that women's career success depends not only on IQ but also on other factors such as environment and personality and suggests four favoring contextual factors for them to translate their talent into career success: (1) above-average intelligence, contextual intelligence, and/or special talent; (2) personality traits such as motivation, creativity, and patience; (3) environmental factors such as family and peer support, timing, and opportunities; and (4) perceived social importance, that is, women's existing desire to use their talent for society. There are no findings on this for men, which is why only women are discussed here.

Results on the critical incidents and work-life compromise variables are available for only a small sample (*N* = 11) of gifted women (Tirri and Koro-Ljungberg, 2002). Critical incidents here are decisions made to achieve success in their careers. Gifted women describe the following critical incidents in their careers: study abroad, choosing the right domain, building one's interest within the field, and previous work experience. Gifted women also make trade-offs—decisions when there are two simultaneous events—regarding career most frequently in the area of career choice (e.g., choosing a career that allows family planning), completing non-motivating tasks (e.g., completing a non-fulfilling degree), completing tasks that are not of primary interest, focusing on one's field (e.g., completing administrative tasks or supervising doctoral students to advance one's career), and choice of job (e.g., returning to academia after working in industry; Tirri and Koro-Ljungberg, 2002). The study shows that even gifted women make tradeoffs to succeed in their careers and, as in previous studies, family planning is an important factor in the career trajectories of gifted women.

Setting aside selective samples of only female scientists (Tirri and Koro-Ljungberg, 2002), gifted men have higher career success in STEM than women. They have significantly more publications and more often have a patent (Lubinski et al., 2014). Career success, however, appears to depend on achievement level: Gifted individuals with a higher SAT-M score (best quartile) are significantly more likely to hold a patent or professorship at a top 50 U.S. university than gifted individuals with a low SAT-M score (lowest quartile; Wai et al., 2005). It seems that even in the group of the most intelligent, an even higher intelligence leads to higher success.

Overall, the results show that gifted individuals sometimes perform exceptionally well at work, with success measured mostly in academia. In this regard, gifted men are more successful at work than women.

### Personality and Behavior

The theme personality and behavior includes information about procrastination, social relationships, ambition and job-related personality.

Subotnik et al. (1999) show that about half of the gifted respondents procrastinate at work. Gifted women procrastinate to spend more time with family or for social activities. Gifted men procrastinate to find time for creativity inside and outside of paid work.

Gifted women describe themselves as sensitive. Social relationships are the most important component for them in their profession. At the same time, however, they also describe them as their greatest weakness (Schuster, 1990).

Regarding ambition, significantly more gifted men than gifted women show above-average ambition in excellence in their work and financial gain (Oden, 1968).

In comparison to non-gifted individuals, gifted individuals report higher ambition than non-gifted individuals in the area of excellence in work (80% of males and 70.5% of females). In comparison, scores are significantly lower in the areas of recognition for achievements (35.9% of males and 28.8% of females), further career advancement (40.4% of males and 34.6% of females), and financial gain (33% of males and 22.3% of females; Oden, 1968).

Hossiep et al. (2012) show for a sample of Mensa members that they differ from non-gifted individuals in terms of job-related personality. They rate themselves weaker than the comparison group in terms of professional orientation (achievement motivation, leadership motivation), work behavior (flexibility, action orientation), social competencies (sensitivity, sociability, team orientation, assertiveness), and psychological constitution. No differences exist in the conscientiousness facet of work behavior. The lower leadership motivation would suggest that gifted individuals are less likely to aspire to leadership positions than non-gifted individuals. However, this is in contrast to the findings reported by other authors, according to which gifted individuals are more likely to hold positions with personnel management responsibility (Schlegler et al., 2018, p. 673; Schuster, 1993). The poorer assessment of social skills reflects the prevailing stereotypes about gifted people (Baudson, 2016, p. 2; Baudson and Preckel, 2013, p. 38). The study by Hossiep et al. (2012) also shows that gifted people assess themselves worse or at least more critically in many facets than non-gifted people. However, it remains unclear from the quantitative survey what reasons gifted individuals have for this. Perhaps gifted people are generally more critical of themselves, have had bad experiences with colleagues in this regard, or the attribution of stereotypes already leads to a worse self-assessment. It also remains questionable whether this poorer assessment would also be shared by others, such as colleagues, superiors, or employees.

The results suggest that gifted individuals are interested in excellent performance, but not so much in career advancement. Moreover, they indicate social relationships at work as one of their weaknesses.

### Satisfaction

The theme satisfaction includes information about job satisfaction, work as a factor influencing life satisfaction, joy from working, and the meaningfulness of work.

Job satisfaction is examined in 13 studies using various constructs (including satisfaction with career, satisfaction with income-generating work, satisfaction with career success). Table 8 shows that gifted individuals are satisfied with their jobs (mean scores of 0.41–0.92 or proportion of satisfied individuals 55–91%). There are clear differences concerning occupational status; specifically, gifted people with higher occupational status are more satisfied (Terman and Oden, 1967; Holahan, 1981; Holahan and Sears, 1995). Regarding differences based on educational attainment, it is reported that gifted women with high levels of education (operationalized as having completed a graduate degree) perceive significantly higher levels of job satisfaction than gifted women with lower levels of education (here, with and without college degrees; Tomlinson-Keasey and Keasey, 1993). One possible explanation for both findings is that gifted individuals with higher occupational status and/or higher levels of education, one the one hand, have greater occupational success, and. one the other hand, are presumably better able to realize their intellectual potential. These abilities in turn lead to satisfaction.

**Table 8:** Job satisfaction by gender, occupational status, and giftedness.

**Satisfaction with**	**Group**	**Mean rescaled (0–1)/ percentage satisfied**	**References**
Occupation	Men and women	>0.86/–	Subotnik et al., 1989
	Men	0.86/86%	Terman and Oden, 1967
	Women (total)	0.88/91%	
	Homemakers	0.88/–	
	Part-time employees	0.84/–	
	Full-time employees, status group 1 (of 2)	0.92/–	
	Full-time employees, status group 2 (of 2)	0.84/–	
	Women with graduate studies	0.53/–	Tomlinson-Keasey and Keasey, 1993
	Women with a college degree	0.41/–	
	Women without a college degree	0.44/–	
	Men and women	0.69/-	Persson, 2009
	Gifted	0.53/-	Siekańska and Sekowski, 2006
	Non-gifted	0.47/-	
Income-generating work	Men (total)	0.87/90%	Holahan and Sears, 1995
	Status group 1 (of 3)	0.89/93%	
	Status group 2 (of 3)	0.85/88%	
	Status group 3 (of 3)	0.81/83%	
	Women (total, including homemakers)	0.89/89%	
	Status group 1 (of 2)	0.92/96%	
	Status group 2 (of 2)	0.82/82%	
Income work (lifetime satisfaction)	Housewives	0.82/-	Holahan, 1981
	Women status group 1 (of 2)	0.91/–	
	Women status group 2 (of 2)	0.83 /	
Career	Gifted	-/63–67%	Benbow et al., 2000
	Talent-search participants	-/55–66%	Ferriman et al., 2009
	Graduate students	-/57–71%	
Success in the professional career	Men	0.82/-	Lubinski et al., 2014^a^
	Women	0.81/–	
The current direction of the professional career	Men	0.78/-	Lubinski et al., 2014
	Women	0.79/–	
Present feelings about work	Housewives	0.87/-	Holahan, 1981
	Women status group 1 (of 2)	0.88/-	
	Women status group 2 (of 2)	0.80/-	

*^a^Also contains the results of Lubinski et al. (2006)*.

Two studies also examine how satisfied gifted individuals are with certain aspects of the profession (see Table 9). Gifted individuals cite creativity, learning, stimulation, personal growth, friendly relationships with people at work and administration, organization, and pride in work done as the most important sources of job satisfaction. However, it remains unclear what exactly is understood by these aspects (Holahan and Sears, 1995). Among gifted women, those with higher occupational status are found to experience the most satisfaction from helping relationships. Gifted women with lower occupational status gain the most satisfaction from financial gain through work, and they report greater satisfaction from friendly relationships at work (Holahan, 1981). However, this contradicts more recent findings for men and women, according to which organizational fit rather than external conditions are most relevant for satisfaction (Siekańska and Sekowski, 2006). Reasons for dissatisfaction among gifted men and women include inappropriate or unchallenging tasks, unsuitable supervisors, difficulties with colleagues, resignation, and alienation (Persson, 2009).

**Table 9:** Satisfaction with partial aspects of the job.

**Satisfaction with the aspect**	**Group**	**Mean rescaled (0–1)**	**References**
Helping	Housewives	0.25	Holahan, 1981
	Women status group 1 (of 2)	0.41	
	Women status group 2 (of 2)	0.34	
Friendship	Housewives	0.34	
	Women status group 1 (of 2)	0.38	
	Women status group 2 (of 2)	0.53	
Financial gain	Housewives	0.10	
	Women status group 1 (of 2)	0.31	
	Women status group 2 (of 2)	0.42	
Income	Gifted	0.61	Persson, 2009
Employers use the full potential		0.61	
Promotion possibility		0.61	
Freedom at work		0.74	
Colleagues quality		0.74	
Scope of responsibility assigned by the employer		0.74	

The Terman studies examine whether job satisfaction correlates with personality and external factors. For gifted individuals (males and females), job satisfaction is weakly to moderately positively correlated with income and motivation (Holahan and Sears, 1995). Detailed research for men shows that job satisfaction is weakly to moderately positively correlated with occupational success, chosen occupation, occupational status, income, the importance of money, education (training), the importance of school, persistence, ambition, affiliation, health, and vitality (Sears, 1977; Holahan and Sears, 1995). Besides, moderate correlations are shown with the condition that the person has lived according to his or her potential (assessment at the end of working life; Sears, 1977).

However, a closer look at the importance of a career for life satisfaction reveals heterogeneous results for men. First, it is assumed that family is more important for gifted people (men and women) than work and career (Sears, 1977; Lubinski et al., 2014). Especially for men, profession is a more important factor for than friendships, cultural life, and commitment to society (Sears, 1977). However, Oden (1968) and Subotnik et al. (1989) show for their samples that gifted men experience the greatest satisfaction from work itself, ahead of family aspects such as marriage and children.

A heterogeneous picture also emerges for women, depending on the time of data collection. In the Terman sample, occupation clears up most of the variance in life satisfaction (Sears and Barbee, 1977). However, gifted women experience the greatest life satisfaction from family (children and marriage) than from avocational activities, and only then from work itself. For women, however, the importance of the individual areas of life depends strongly on their occupational status. While women without employment or with part-time employment experience the greatest satisfaction from family, the work factor is the most important for women with full-time employment (Oden, 1968). However, in a later survey, gifted women experience most life satisfaction from work and children (Subotnik et al., 1989).

Several studies look at gender differences in job satisfaction among gifted individuals and come to different conclusions. Three studies show that there are no differences between men and women in job satisfaction (Subotnik et al., 1989; Benbow et al., 2000) and satisfaction with direction or career success (Lubinski et al., 2014). However, one of the Terman studies concludes that gifted males experience higher job satisfaction than gifted females (Holahan et al., 1999).

Looking at talent-search participants in the SMPY study, men are significantly more satisfied with their lives than women, while there are no gender differences among graduate students (Ferriman et al., 2009).

Siekańska and Sekowski (2006) examine the differences between gifted individuals and a control group of non-gifted individuals. According to this study, job satisfaction is significantly higher among the gifted than among the control group. Both groups rate satisfaction with financial aspects (payment, compensation system) as the lowest. Among the gifted group, work is more often related to their interests. They are also more often able to use their skills, dispositions, and expertise at work and improve their qualifications at work. They are more likely to see work as a source of pleasure/enjoyment than the non-gifted individuals in the control group. However, gifted individuals are less likely to perceive a good atmosphere in the team (Siekańska and Sekowski, 2006). This result suggests that gifted individuals seek occupations that match their abilities and interests. Satisfaction then seems to be rooted in the work itself and less in external circumstances.

A study by the Marburg Giftedness Project also shows results on the influence of occupation on life satisfaction in gifted people compared to non-gifted people (control group). According to this study, among gifted individuals, work is the only predictor of life satisfaction. By contrast, for non-gifted individuals, the self and friends are also predictors (Wirthwein and Rost, 2011). This finding suggests that gifted individuals place more importance on work in their lives than non-gifted individuals. According to the authors of the study, this could be because gifted people have more challenging jobs and spend more time in their lives working than non-gifted people (Wirthwein and Rost, 2011).

A comparison between gifted, high-achieving, and non-gifted individuals shows that gifted individuals feel less meaningfulness in their work than high-achievers and a control group of non-gifted individuals. Furthermore, for gifted people and high achievers, the meaningful work variable explains and influences the meaningfulness (of life) outcome variable, with meaningful work even being the most important predictor for high achievers (Pollet and Schnell, 2017). The result suggests that for high achievers, who by definition have high levels of success at work, work has a stronger impact on the meaningfulness of life. Moreover, of the three groups, gifted individuals experience the least meaningfulness at work. However, this contradicts the results on life satisfaction reported by Wirthwein and Rost (2011).

Gifted individuals experience less joy from working than high achievers and non-gifted individuals. The joy of working correlates slightly negatively with fluid intelligence (Pollet and Schnell, 2017). This relationship could be because at a higher level of intelligence, individuals are less cognitively challenged by the same work and therefore derive less pleasure from their work. Moreover, for gifted people, the joy of working predicts subjective wellbeing. This is not the case for high achievers (Pollet and Schnell, 2017). These results are consistent with findings from the Marburg Giftedness Project (Wirthwein and Rost, 2011). Overall, the results suggest that gifted individuals are more influenced by the joy of working. Negative feelings at work also have a negative effect on non-work-related subjective wellbeing.

In general, gifted individuals are satisfied with their work. However, the degree of job satisfaction varies from individual to individual and depends on whether the person subjectively feels that he or she is doing justice to his or her intellectual abilities (intellectual potential is being exploited). Besides, external factors (compensation) and personality (ambition, persistence), as well as social relationships at work, have an impact on the job satisfaction of gifted individuals. The analyzed studies show that occupation is an important factor in the life satisfaction of gifted individuals. However, gifted men experience life satisfaction more strongly from their occupation than women (Holahan, 1985). For women, the importance of occupation for life satisfaction is also influenced by the prevailing role model at the time of data collection and occupational status (employed or housewife). At younger ages and with a higher occupational status, occupation is more important for life satisfaction among gifted women.

### Organization

Information about organizational fit and desires of the gifted individuals for the organization are only reported by Shareef (2015) who uses a self-assessment for the data collection.

Gifted individuals with high self-concept (high self-assessment in likability, morale, task performance, giftedness, power, and vulnerability) show lower levels of organizational fit. In this context, likability, morality, and giftedness, but not task performance, power, or vulnerability, correlate with the fit. Thus, individuals who assess themselves more critically perceive a higher fit with the employer than individuals with a more positive self-assessment. One possible explanation is that individuals with a high self-concept also have higher self-confidence and self-assurance, which leads to the fact that the individuals can also live with a lower fit and therefore do not adapt more because they do not make their wellbeing dependent on the fit. The model shows that work experience acts as a moderator between self-assessment and fit, but this is not true for social support (Shareef, 2015).

Gifted individuals also express desires they have for the organization in which they work. A portion would like to see changes in the areas of organizational culture (alignment of organizational goals with personal goals, greater autonomy, expanded communication, more flexible organization, better collaboration within the organization, more freedom, transparency, appreciation), strategy (stronger strategic orientation, stronger orientation to strategy) and personnel development (more development opportunities, mentoring, more support for employees; Shareef, 2015). They demand more freedom and autonomy concerning their activities. This fits with other findings that gifted individuals are more often self-employed than non-gifted individuals (Schlegler et al., 2018).

### Influence of Giftedness on the Profession

Only Shareef (2015) examines what gifted individuals believe the impact of giftedness has on their professional situation (there is no matching with a view of colleagues, managers, subordinate employees, etc.). The perceived influence of giftedness is highly individual. While some participants feel no influence, others experience a variety of influences. General statements are therefore difficult to make. However, it can be seen that the perceived influences can be both negative (participants experience boredom and have difficulty communicating) and positive (participants have higher self-confidence and faster comprehension). As a consequence of the influence, gifted individuals adjust their communication with non-gifted individuals.

### Miscellaneous

Approximately 25% of one sample of gifted women cite organizational skills as one of their professional competencies (Hollinger and Fleming, 1992). A large proportion of another sample (75%) says they find their greatest intellectual challenge on the job (Schuster, 1993). In the study by Tomlinson-Keasey and Keasey (1993), the majority of gifted women report that they have fairly well realized their intellectual potential.

## Discussion

### Summary of the Main Findings

This paper is a systematic literature review of the occupational situation of gifted adults. It aims to present and link existing findings on the employment situation, job satisfaction, and any differences based on gender and giftedness. It looks at the subjective experiences of a group of individuals and highlights findings on prevailing stereotypes about gifted individuals such as their tendency to experience emotional and social problems. For this purpose, 40 primary studies with a total of 22 variables were analyzed. The results from several studies could be evaluated and compared for 11 variables, while the results from only one study per variable were available for the other half of the variables. The variables could be grouped into the following categories: (a) employment situation, (b) career, (c) personality and behavior, (d) satisfaction, (e) organization, (f) influence of giftedness on the profession and (g) miscellaneous. Most of the studies included in the review were from the United States. Indeed, there are hardly any European studies and no studies from other parts of the world on the research questions, at least in German or English.

The results show first of all that the examined gifted people are mostly successful in their professional situation. The majority is employed and shows a high occupational status. In addition, the participants work in various occupational fields, but more frequently in occupational fields that offer cognitive rather than manual tasks and therefore tend to challenge the gainfully employed more cognitively. Besides, a high level of education is required for many of the jobs, which gifted individuals largely have (Sparfeldt, 2006). In addition, they frequently hold positions with personnel responsibility. This confirms previous results of meta-analyses on the occupational situation of highly intelligent individuals (Kuncel et al., 2004; Schmidt and Hunter, 2004; Schmidt, 2009; Herrnstein and Murray, 2010; Pässler et al., 2015; Schmidt et al., 2016; Murtza et al., 2020). It seems that gifted people also realize their potential at work. Gifted people usually show a high level of job satisfaction which initially contradicts common stereotypes (Plucker and Levy, 2001). Job satisfaction is most strongly influenced by autonomy and responsibility in the job and the relationship with colleagues. In general, it can be said that gifted individuals are more satisfied in their jobs if they take a high degree of responsibility for the assigned tasks, can organize their activities freely and independently, and have good social contacts in the professional context. The needs of competence, autonomy, and relatedness are considered basic human needs in self-determination theory (Ryan and Deci, 2017). Comparable to non-gifted individuals, gifted individuals also seem to have the desire to satisfy the three basic needs in the professional context. The influence on giftedness on the profession seems to be individual for each person. Difficulties of gifted people at work are only mentioned in a few studies (e.g., Shareef, 2015), whereby this assessment is mainly subjective and is described as such by the gifted individuals themselves. Difficulties for gifted individuals seem to be primarily in communicating with non-gifted individuals, as gifted individuals indicate that they use adapted communication.

There are various differences between the occupational situations of gifted men and women. At first, gifted women are more family-oriented than gifted men (prefer time for family and friends, shorter working hours, greater flexibility), while the latter prefer job-related aspects more strongly (salary, challenging work, freedom to choose tasks themselves). These findings are analogous to findings for the general population (Hakim, 1991, 2002). Besides, the proportion of men in social professions is lower as for women, analogous to the overall population (Deutscher Bundestag, 2012). Moreover, women work fewer hours than men which can also be seen in the total population (OECD, 2018). The tendency of women to work fewer hours per week can thus be observed independently of giftedness. This is certainly also a reason why gifted women earn less on average than gifted men. The majority of studies show that the gender pay gap that exists in the overall population also exists among gifted people. Besides the working hours it can also be explained by the higher part-time rate among women (OECD, 2017; U.S. Bureau of Labor Statistics, 2019), although it also exists in the comparison of full-time employees (Blau and Kahn, 2016; U.S. Bureau of Labor Statistics, 2019). In general, it can be stated that men—measured by objective criteria—are more successful in their professions, especially in academic professions, although this is also independent of giftedness in the population. In the United States, e.g., 32% of professorships are held by women, which is significantly less frequently than by men (Warner et al., 2018).

Most differences in the gifted population due to gender can be traced back to the role model of women existing at the time of data collection. For example, the Terman study was initiated as early as 1921/22 (Baudson, 2008). Even the methodology shows how strongly the careers of women have been partly dependent on the family (classification in employed or housewife), while for men, a traditional distinction has been made between employed and unemployed. The differences between men and women are to be expected because—until today—in most cultures and regions, women take on a more dominant role in child-rearing and therefore forego gainful employment more often or do so only to a lesser extent than men (Schulte, 2002; Neumaier, 2019). However, a positive trend can also be seen in the population over time, with the proportion of women in employment rising steadily (Kiss, 2020). Due to the changing role models of men and women and their possible roles in families, as men these classifications should be looked at again today and more complex career patterns of men should also be examined.

Differences can also be found to some extent between gifted and non-gifted individuals. Gifted people have a higher occupational status than non-gifted people, are less likely to be unemployed, and have a higher income. They also show higher levels of ambition and job satisfaction. In addition, work is a more important component of life satisfaction for gifted than non-gifted individuals. However, compared with non-gifted individuals, gifted individuals—operationalized in the sample by membership in Mensa—perceive less enjoyment and meaningfulness in work and have a more critical self-assessment of their personality, especially about social skills. They also exhibit lower leadership motivation. However, lower leadership motivation contrasts with the finding that gifted individuals—in this sample also Mensa members—are more likely than non-gifted individuals to occupy leadership positions (Schlegler et al., 2018). The poorer self-assessment of social skills could possibly be explained by social identity theory (SIT). According to SIT, people tend to classify other people into groups (outgroup) and to assign stereotypes to them that distinguish them from their own group (ingroup) (Tajfel and Turner, 1979). Two German studies show that two-thirds of the population have negative stereotypes about gifted people (Baudson, 2016) and, moreover, that gifted people (Mensa members) see themselves as a minority (Baudson and Ziemes, 2016). The question, therefore, arises as to whether the gifted individuals identify with their role as gifted and thereupon also map the existing, mostly ascribed, stereotypes onto their own person and judge themselves accordingly and possibly also act accordingly. However, it remains unclear whether this applies to gifted individuals in general or only the specific samples of Mensa members.

### Limitations

The studies included in the review show some limitations from which future research approaches can be developed. First, more than half of the studies (*N* = 21) were conducted more than 20 years ago at the time of the review (Terman and Oden, 1959, 1967; Oden, 1968; Sears, 1977; Sears and Barbee, 1977; Holahan, 1981, 1985, 1994; Kaufmann et al., 1986; Subotnik et al., 1989, 1999; Schuster, 1990, 1993; Tomlinson-Keasey, 1990; Hollinger and Fleming, 1992; Arnold, 1993; Tomlinson-Keasey and Keasey, 1993; Holahan and Sears, 1995; Kastberg and Miller, 1996; Reis, 1996; Holahan et al., 1999). The Terman study in particular (Terman and Oden, 1959, 1967; Oden, 1968; Sears, 1977; Sears and Barbee, 1977; Holahan, 1981, 1985, 1994, 2003; Tomlinson-Keasey, 1990; Tomlinson-Keasey and Keasey, 1993; Holahan and Sears, 1995; Holahan et al., 1999) has been frequently criticized for methodological and ethical shortcomings (Giger, 2009). The time of data collection, as well as the social situation at that time, seem to be important. Thus, even in longitudinal studies, the results differ among the respective survey dates. Larger time-related differences are particularly evident among women. While the majority worked as housewives in the mid-twentieth century and their focus was on raising children, the importance of a career has increased for many women over the years. This can be seen, for example, in the complex career paths of women as well as the retrospective desire to have had a greater focus on career after all. However, there were also women in the mid- twentieth century who placed an emphasis on career and thus had different experiences than women of the same age who chose to be homemakers. After all, the social role image of women has changed considerably in the last century and women make up a higher proportion of the workforce in the overall population (Wernhart and Neuwirth, 2007; OECD, 2017).

Second, only a few studies make comparisons to control groups of non-gifted individuals, so that overall hardly any conclusions can be drawn about the differences between gifted and non-gifted workers. So far, the author is aware of only one study, the Marburg Giftedness Project, which addresses both of these biases by using an unselected sample of gifted and non-gifted students (Rost, 2009). Thus, no conclusions can be drawn about the extent to which the results found—for example, gender differences—are primarily related to giftedness or apply to other populations as well.

Third, in all studies except Wirthwein and Rost (2011), there is the possibility of sample bias because the selection of gifted individuals was often done by self-selection (e.g., membership in a gifted association) or by teacher nomination and school achievement tests. This means that underachievers, that is, individuals with potential but who do not translate it into achievement, could not be captured, as they are usually excluded in these selections. In the case of selection by membership in a gifted association, underachievers could in principle be included in the respective sample. However, a prerequisite for membership in a gifted association is that the giftedness is known to the person and that he or she wants to deal with it actively. This in turn leads to a bias, because often gifted people with difficulties seek an exchange with like-minded people in such an association and they are thus supposedly different from other gifted people. Despite the criticisms, the selection procedures used seem feasible, because extensive surveys with intelligence diagnostics would have to be conducted for an unselected sample that also includes undetected gifted individuals, assuming a proportion of gifted individuals of 2% of the total population. It also remains unclear what influence the support of gifted individuals identified in childhood and adolescence has on their later occupational situation, because hardly any samples were drawn only in adulthood and, except for the Marburg Giftedness Project, the academic support of gifted children and adolescents was usually part of the studies. This could also lead to a bias of the results in adulthood. It would therefore be desirable to repeat studies with unselected samples and comparison groups to find out which results can be attributed to giftedness and which are individual and dependent on the composition of the sample.

Fourth, some studies show limitations in the presentation of results. Their results are only presented in diagrams without reporting the numeric results (Lubinski et al., 2006, 2014; Ferriman et al., 2009), which makes an evaluation very difficult. To facilitate future reviews, it would be desirable that the values are also presented in tabular form with the indication of standard deviations, the results of statistical tests, and, if possible, effect sizes.

This review also identifies existing research gaps. First, it would be interesting to see a more current survey on the variables considered in the Terman study to determine whether the results still hold in a more recent context, or whether the results are more dependent on the survey period than on the giftedness of the participants. It would be expected that the employment situation of women, in particular, has developed away from a majority activity as housewives and toward a career of their own. It would be expected that this social trend would also be evident among the gifted group. Second, most of the results presented employed quantitative survey methods with large samples. Only a small proportion of the studies used a qualitative approach (e.g., Kastberg and Miller, 1996; Tirri and Koro-Ljungberg, 2002). In the future, existing results from large samples could be examined in more detail and depth using qualitative research methods and a subsample. This would be desirable, for example, in studies like the one by Hossiep et al. (2012), in which the low leadership motivation of gifted individuals is discussed. A qualitative approach could also clarify why there is a lower leadership motivation among gifted people, whether there are similar reasons within the group for this, and whether the lower leadership motivation has an influence on taking on leadership positions by gifted people. Moreover, complementing the findings of Shareef (2015), a future research question could be why gifted individuals adapt their communication at work and what methods and measures they consciously and unconsciously use to do so. Besides, aspects of job satisfaction could be investigated qualitatively, for example by interviewing gifted individuals to find out how an optimal work situation for gifted should be designed to lead to high job satisfaction. It could be investigated whether these situations show patterns due to giftedness or are individually different.

In addition to the limitations of the reviewed studies, the review itself is restricted due to some limitations. First, the search was only conducted in German and English due to the author's language skills. Publications in other languages could not be recorded as a result. Second, the used definition of giftedness is narrow. Only academic and intellectual giftedness were examined according to both performance and potential definitions, while, for example, musical and athletic giftedness were excluded from the review. Thus, based on the definition of giftedness in adults by Subotnik et al. (2011) one could argue that the focus of the review is on highly intelligent rather than gifted adults. However, a focus on academic and intellectual giftedness is justifiable because, especially in the European context, the potential definition is also used (Rost, 2009) and other forms of giftedness are still difficult or impossible to measure (Sparfeldt, 2006; Sparfeldt et al., 2009).

## Conclusions

This review highlights some implications for practice. First, it can serve as an awareness-raiser for human resource managers and executives. Gifted individuals are present—albeit in varying proportions—in all occupational fields and status groups. Therefore, managers should be sensitized to the presence of giftedness. Furthermore, many stereotypical assumptions about gifted individuals, such as a stronger tendency toward social and emotional problems, could not be confirmed in the professional context. Therefore, one implication would be those non-gifted individuals, both colleagues, and managers, reflect on their stereotypical assumptions about gifted individuals and refrain from attributing them to gifted individuals. Furthermore, the results show that gifted individuals are successful at work and thus may contribute to the success of the organization. Hiring a gifted person could therefore have a positive impact on the organization.

Overall, this review provides a comprehensive overview of a hitherto poorly studied subfield of giftedness research and provides avenues for further investigation.

## Data Availability Statement

The original contributions presented in the study are included in the article/Supplementary Material, further inquiries can be directed to the corresponding author.

## Author Contributions

The author confirms sole responsibility for the following: study conception and design, data collection, analysis and interpretation of results, and manuscript preparation.

## Funding

Open Access Publication Fund was provided by the Goethe University Library.

## Conflict of Interest

The author declares that the research was conducted in the absence of any commercial or financial relationships that could be construed as a potential conflict of interest.

## Publisher's Note

All claims expressed in this article are solely those of the authors and do not necessarily represent those of their affiliated organizations, or those of the publisher, the editors and the reviewers. Any product that may be evaluated in this article, or claim that may be made by its manufacturer, is not guaranteed or endorsed by the publisher.
