# Supplementary material for: Systematic Literature Review: Professional Situation of Gifted Adults
Source: Front Psychol. 2022 May 18;13:736487. doi: 10.3389/fpsyg.2022.736487 (PMC9158468; doi:10.3389/fpsyg.2022.736487)
Supplement: Supplementary file 1 [file Data_Sheet_1.docx]

Table 1: Overview of the studies with the included variables

|  | **Employment situation** | **Occupational status** | **Occupational field** | **Income** | **Working time** | **Career goals** | **Career paths and patterns** | **Professional success** | **Job satisfaction** | **Work preferences** | **Work as a factor influencing life**  **satisfaction** | **Job-related personality** | **Ambition** | **Social relationships** | **Joy of working** | **Meaningfulness of work** | **Critical incidents and compromises in**  **professional life** | **Procrastination** | **Influence of giftedness on the profession** | **Organizational fit** | **Desires for the organization** | **Miscellaneous** |
| --- | --- | --- | --- | --- | --- | --- | --- | --- | --- | --- | --- | --- | --- | --- | --- | --- | --- | --- | --- | --- | --- | --- |
| Arnold (1993) |  |  | x |  |  |  |  | x |  |  |  |  |  |  |  |  |  |  |  |  |  |  |
| Benbow et al. (2000) |  |  | x | x | x |  |  |  | x | x |  |  |  |  |  |  |  |  |  |  |  |  |
| Feist (2006) |  |  | x |  |  |  |  | x |  |  |  |  |  |  |  |  |  |  |  |  |  |  |
| Ferriman et al. (2009) |  |  |  |  |  |  |  |  | x | x | x |  |  |  |  |  |  |  |  |  |  |  |
| Holahan (1981) |  |  |  |  |  |  |  |  | x |  |  |  |  |  |  |  |  |  |  |  |  |  |
| Holahan (1985) |  |  |  |  |  |  |  | x |  |  | x |  |  |  |  |  |  |  |  |  |  |  |
| Holahan (1994) |  |  | x |  |  |  | x |  |  |  |  |  |  |  |  |  |  |  |  |  |  |  |
| Holahan (2003) |  |  |  |  |  |  | x |  |  |  |  |  |  |  |  |  |  |  |  |  |  |  |
| Holahan et al. (1999) |  |  |  |  |  |  |  |  | x |  |  |  |  |  |  |  |  |  |  |  |  |  |
| Holahan and Sears (1995) |  | x | x | x |  | x | x |  | x |  |  |  |  |  |  |  |  |  |  |  |  |  |
| Hollinger and Fleming (1992) |  | x |  |  |  |  |  |  |  |  |  |  |  |  |  |  |  |  |  |  |  | x |
| Hossiep et al. (2012) |  |  |  |  |  |  |  |  |  |  |  | x |  |  |  |  |  |  |  |  |  |  |
| Kastberg and Miller (1996) |  |  | x |  |  |  |  |  |  |  |  |  |  |  |  |  |  |  |  |  |  |  |
| Kaufmann et al. (1986) |  |  | x | x |  |  |  |  |  |  |  |  |  |  |  |  |  |  |  |  |  |  |

|  | **Employment situation** | **Occupational status** | **Occupational field** | **Income** | **Working time** | **Career goals** | **Career paths and patterns** | **Professional success** | **Job satisfaction** | **Work preferences** | **Work as a factor influencing life satisfaction** | **Job-related personality** | **Ambition** | **Social relationships** | **Joy of working** | **Meaningfulness of work** | **Critical incidents and compromises in professional life** | **Procrastination** | **Influence of giftedness on the profession** | **Organizational fit** | **Desires for the organization** | **Miscellaneous** |
| --- | --- | --- | --- | --- | --- | --- | --- | --- | --- | --- | --- | --- | --- | --- | --- | --- | --- | --- | --- | --- | --- | --- |
| Kell et al. (2013) |  |  | x | x |  |  |  | x |  |  |  |  |  |  |  |  |  |  |  |  |  |  |
| Lubinski et al. (2014) | x | x | x | x | x |  |  | x | x | x | x |  |  |  |  |  |  |  |  |  |  |  |
| Lubinski et al. (2006) |  |  | x | x | x |  |  |  | x |  |  |  |  |  |  |  |  |  |  |  |  |  |
| Makel et al. (2016) |  |  | x |  |  |  |  | x |  |  |  |  |  |  |  |  |  |  |  |  |  |  |
| Oden (1968) |  |  | x | x |  |  |  | x |  |  | x |  | x |  |  |  |  |  |  |  |  |  |
| Park et al. (2008) |  |  |  |  |  |  |  | x |  |  |  |  |  |  |  |  |  |  |  |  |  |  |
| Perrone et al. (2004) |  |  |  |  |  | x |  |  |  |  |  |  |  |  |  |  |  |  |  |  |  |  |
| Persson (2009) |  |  | x |  |  |  |  |  | x |  |  |  |  |  |  |  |  |  |  |  |  |  |
| Pollet and Schnell (2017) | x | x | x |  |  |  |  |  |  |  |  |  |  |  | x | x |  |  |  |  |  |  |
| Reis (1996) |  |  |  |  |  |  |  | x |  |  |  |  |  |  |  |  |  |  |  |  |  |  |
| Schuster (1990) | x |  | x | x |  |  |  | x |  |  |  |  |  | x |  |  |  |  |  |  |  |  |
| Schuster (1993) | x |  | x |  |  |  |  |  |  | x |  |  |  |  |  |  |  |  |  |  |  | x |
| P. S. Sears and Barbee (1977) | x | x |  |  |  |  | x |  |  |  | x |  |  |  |  |  |  |  |  |  |  |  |
| R. R. Sears (1977) |  |  |  |  |  |  |  |  | x |  | x |  |  |  |  |  |  |  |  |  |  |  |

|  | **Employment situation** | **Occupational status** | **Occupational field** | **Income** | **Working time** | **Career goals** | **Career paths and patterns** | **Professional success** | **Job satisfaction** | **Work preferences** | **Work as a factor influencing life satisfaction** | **Job-related personality** | **Ambition** | **Social relationships** | **Joy of working** | **Meaningfulness of work** | **Critical incidents and compromises in professional life** | **Procrastination** | **Influence of giftedness on the profession** | **Organizational fit** | **Desires for the organization** | **Miscellaneous** |
| --- | --- | --- | --- | --- | --- | --- | --- | --- | --- | --- | --- | --- | --- | --- | --- | --- | --- | --- | --- | --- | --- | --- |
| Shareef (2015) |  | x |  | x |  |  |  |  |  |  |  |  |  |  |  |  |  |  | x | x | x |  |
| Siekańska and Sękowski (2006) | x |  | x |  |  |  |  |  | x |  |  |  |  |  |  |  |  |  |  |  |  |  |
| Subotnik et al. (1989) |  |  | x | x |  |  |  |  | x |  | x |  |  |  |  |  |  |  |  |  |  |  |
| Subotnik et al. (1999) |  |  | x |  |  |  |  |  |  |  |  |  |  |  |  |  |  | x |  |  |  |  |
| Terman and Oden (1959) | x | x | x | x |  |  |  |  |  |  |  |  |  |  |  |  |  |  |  |  |  |  |
| Terman and Oden (1967) | x | x | x | x |  |  |  |  | x |  |  |  |  |  |  |  |  |  |  |  |  |  |
| Tirri and Koro-Ljungberg (2002) |  |  |  |  |  |  |  | x |  |  |  |  |  |  |  |  | x |  |  |  |  |  |
| Tomlinson-Keasey (1990) |  |  |  |  |  |  | x |  |  |  |  |  |  |  |  |  |  |  |  |  |  |  |
| Tomlinson-Keasey and Keasey (1993) |  | x |  | x |  |  | x |  | x |  |  |  |  |  |  |  |  |  |  |  |  | x |
| Wai et al. (2005) |  |  |  | x |  |  |  | x |  |  |  |  |  |  |  |  |  |  |  |  |  |  |
| Wai et al. (2010) |  |  |  |  |  |  |  | x |  |  |  |  |  |  |  |  |  |  |  |  |  |  |
| Wirthwein and Rost (2011) |  |  |  |  |  |  |  |  |  |  | x |  |  |  |  |  |  |  |  |  |  |  |
